# Genome Sequences of *Mannheimia haemolytica* Serotype A1 Strains D153 and D193 from Bovine Pneumonia

**DOI:** 10.1128/genomeA.00848-13

**Published:** 2013-10-17

**Authors:** Melissa J. Hauglund, Fred M. Tatum, Darrell O. Bayles, Samuel K. Maheswaran, Robert E. Briggs

**Affiliations:** USDA, ARS, National Animal Disease Center, Ames, Iowa, USAa; Department of Veterinary and Biomedical Sciences, University of Minnesota, St. Paul, Minnesota, USAb

## Abstract

Here we report two genome sequences, one complete and one draft, from virulent bovine strains of *Mannheimia haemolytica* serotype A1 recovered prior to the field usage of modern antimicrobial drugs.

## GENOME ANNOUNCEMENT

*Mannheimia haemolytica* is a facultative respiratory pathogen of ruminants. Among cattle, serotypes A1, A2, and A6 colonize and are commonly recovered from the nasopharynx (1). Under conditions of concurrent virus infection and/or stress, serotypes 1 and 6 selectively proliferate to become more abundant in these tissues and predominate over serotype A2 (2, 3). In pneumonic disease it is predominantly serotypes A1 and A6 which are recovered from diseased tissues (4). Antimicrobial resistance among bacterial bovine respiratory disease pathogens is of growing concern (5, 6), and multidrug-resistant isolates of *Pasteurella multocida* and *M. haemolytica* have recently been sequenced (7, 8). Isolates D153 and D193 were recovered from pneumonic calf lung in December and January of 1983 to 1984. The genome sequences of these strains will further our understanding of the genetic basis of selective bacterial proliferation in the nasopharynx and provide insight into the acquisition of antimicrobial resistance.

The genome sequencing of *M. haemolytica* strain D153 was achieved using 3 platforms: the Roche (454) GS FLX titanium, resulting in 26-fold coverage; the Illumina GA IIx, resulting in 1,700-fold coverage; and the PacBio RS, resulting in 30-fold coverage. Illumina reads were used to error correct the PacBio reads using CLC-Genomics Workbench v 6.0.2. A hybrid assembly using the CLC software was generated, and the resultant contigs were aligned to an optical map (OpGen, MapSolver software, Gaithersburg, MD) to confirm the assembly and generate a single scaffold. Reiterative alignments of the 454 and corrected PacBio reads of >1 kb against the scaffold, performed using the CLC software, closed all gaps and resulted in a single circular chromosome. The completed D153 genome sequence consists of 2.68 Mb, with a G+C content of 41.04%. The draft genome sequence of *M. haemolytica* D193 was determined using the Roche platform alone, which yielded 39-fold coverage. Assembly against the closed D153 reference genome using the CLC software yielded 50 contigs consisting of 2.68 Mb, with a G+C content of 41.08%, an *N*_50_ of 139,865 bp, and 100% of contigs with lengths of >500 bp.

Annotation of both genome sequences was accomplished with the NCBI Prokaryotic Genome Annotation Pipeline (revision 2.0 and 2.1 for strains D153 and D193, respectively). Strain D153 contained a total of 2,766 genes, including 2,641 predicted protein-encoding genes, 43 frameshifted pseudogenes, 20 rRNA genes, and 62 tRNA genes. Strain D193 contained a total of 2,801 genes, including 2,689 predicted protein-encoding genes, 39 frameshifted pseudogenes, 16 rRNA genes, and 57 tRNA genes. One CRISPR array was detected in each isolate. In contrast to the multiresistant *M. haemolytica* isolate 42548 (7), both strains D153 and D193 lack the genes *aphA1*, *strA*, *strB*, and *sul2*. *tetR* and *tetH* were detected in strain D193 but not D153. Determination of genes potentially involved in selective proliferation in calf nasopharyngeal tissues will require additional genomic sequencing and analysis.

### Nucleotide sequence accession numbers.

The genome sequence of *M. haemolytica* strain D153 has been deposited in GenBank under accession no. CP005972. The genome sequence of *M. haemolytica* D193 has been deposited in GenBank under accession no. ATSY00000000. The version described in this paper is version ATSY01000000.
